# A ferroptosis-related lncRNAs risk score model identifies LINC02178 as a potential regulator of ferroptosis sensitivity in cervical cancer

**DOI:** 10.3389/fonc.2026.1807953

**Published:** 2026-05-08

**Authors:** Huasu Chen, Chaokun She, Zijian Wang, Xiaoxue Huang, Yan Wang, Yafei Zhang, Qianhao Huang, Yudi Rao, Xin Yuan, Zhengfei Gan, Lin Xiong, Kaifeng Wu, He Zha

**Affiliations:** 1 Department of Laboratory Medicine, The Third Affiliated Hospital of Zunyi Medical University (The First People’s Hospital of Zunyi), Zunyi, China; 2 Scientific Research Center, The Third Affiliated Hospital of Zunyi Medical University (The First People’s Hospital of Zunyi), Zunyi, China

**Keywords:** cervical cancer, ferroptosis, HPV, LINC02178, lncRNAs, risk score model

## Abstract

**Background:**

Cervical cancer remains a major global health burden among women. Persistent infection with high-risk human papillomavirus (HR-HPV) is the primary driver of cervical carcinogenesis, yet additional cellular regulatory processes are required for tumor progression. Ferroptosis, an iron-dependent form of regulated cell death, has been implicated in cancer metabolism and therapeutic response. However, the involvement of ferroptosis-related long non-coding RNAs (FRLs) in human papillomavirus (HPV)-associated cervical cancer remains insufficiently understood.

**Methods:**

Transcriptomic and clinical data from The Cancer Genome Atlas (TCGA) and UCSC Xena were analyzed to identify FRLs. A prognostic risk-scoring model (RSM) was constructed using LASSO Cox regression and evaluated by survival analysis and time-dependent receiver operating characteristic (ROC) curves. Expression patterns of selected FRLs were further examined in cervical swab samples with different HPV infection subtypes using quantitative polymerase chain reaction (qPCR). Functional validation was performed in HPV16-positive SiHa cells following small interfering RNA (siRNA)-mediated knockdown of LINC02178, combined with ferroptosis modulation using Erastin or Ferrostatin-1. Cell viability, migration, and ferroptosis-associated biochemical indicators were assessed.

**Results:**

A prognostic model based on 22 FRLs showed stable predictive performance and remained an independent prognostic factor after adjustment for clinical variables. Among these FRLs, LINC02178 emerged as a standout risk factor, exhibiting markedly elevated expression in HPV16-positive tissues and a strong positive correlation with the lipid droplet-associated protein PLIN2—suggesting a potential role in lipid metabolism. *In vitro* experiments demonstrated that LINC02178 knockdown inhibited cell proliferation and migration. Furthermore, silencing LINC02178 increased cellular susceptibility to ferroptosis, as reflected by altered redox status and enhanced oxidative stress, particularly under erastin treatment, evidenced by surging levels of reactive oxygen species (ROS), malondialdehyde (MDA), and ferrous iron accumulation (Fe²^+^) alongside depleted glutathione (GSH). These findings suggest that LINC02178 potentially serves as a critical metabolic shield, contributing to the ability of HPV-infected cells to evade ferroptosis and sustain tumor progression.

## 1 Introduction

Cervical cancer remains a formidable global health crisis, disproportionately affecting women in developing regions where it continues to drive high rates of morbidity and mortality (1). Although the implementation of vaccination programs and routine screening has successfully curbed incidence rates in some populations, the clinical reality for patients diagnosed with advanced or metastatic disease remains grim, with five-year survival rates stalling at disappointingly low levels (2). Central to this pathology is persistent infection with HR-HPV, among which HPV16 distinguishes itself as the most aggressive and prevalent subtype driving tumorigenesis (3). Classically, this malignancy is understood through the action of viral oncoproteins E6 and E7, which do more than merely disrupt cell cycle checkpoints; by degrading p53 and inactivating the Rb pathway, they fundamentally destabilize the host genome and initiate cellular transformation (4). Yet, genomic instability tells only part of the story. Emerging evidence suggests that HPV actively manipulates the cellular environment on a metabolic level, specifically by altering lipid metabolism and ROS signaling. This metabolic reprogramming tilts the oxidative balance, potentially arming tumor cells with resistance against regulated cell death mechanisms—a critical area of adaptation that warrants deeper investigation (5).

First defined in 2012, ferroptosis represents a distinct frontier in our understanding of regulated cell death, fundamentally diverging from the well-mapped landscapes of apoptosis, necrosis, and autophagy (6). Characterized by iron-dependent lipid peroxidation, this process hinges on a delicate balance between cellular iron homeostasis, lipid metabolism, and antioxidant defense systems—a triad that, when disrupted, triggers lethal oxidative damage (7). This metabolic dependency makes ferroptosis particularly relevant in oncology. Tumor cells, driven by a voracious appetite for iron and hyperactive metabolism, are intrinsically susceptible to this form of death; yet, they often evolve sophisticated evasion mechanisms. By upregulating glutathione peroxidase 4 (GPX4) activity or rewiring lipid metabolism, malignant cells can effectively mask this vulnerability, creating a “tolerance” that supports survival under stress (8, 9). Exploiting this duality offers a promising therapeutic strategy, potentially bypassing the resistance mechanisms that plague conventional treatments (10). However, the regulatory networks governing ferroptosis are highly context-dependent. While the pathways in hepatocellular carcinoma or lung cancer are increasingly well-characterized (11, 12), our understanding of ferroptosis in cervical cancer remains fragmented. Specifically, a systematic exploration of how HR-HPV infection—the disease’s primary driver—might manipulate these ferroptotic pathways is largely missing. Bridging this gap is critical, as it stands between our current theoretical understanding and the practical implementation of ferroptosis-targeted therapies for cervical cancer patients.

Long non-coding RNAs (lncRNAs)—transcripts exceeding 200 nucleotides that lack protein-coding potential—have emerged as versatile architects of cellular regulation, orchestrating processes that range from chromatin remodeling to precise transcriptional oversight (13, 14). Recent literature has increasingly underscored their pivotal role in driving tumor metabolic reprogramming and dictating cell death fates (15). In the specific context of ferroptosis, these molecules are not merely bystanders but active participants, fine-tuning the delicate balance between antioxidant defenses, lipid metabolism, and iron homeostasis (16). Mechanistically, many lncRNAs function as competitive endogenous RNAs (ceRNAs) or act as molecular “sponges” for microRNAs, thereby protecting ferroptosis-related transcripts or lipid enzymes from degradation (17). The clinical utility of these regulatory patterns is evident in recent successes, such as the development of FRLs-based RSM in colorectal cancer that effectively stratify patient outcomes (18). Yet, despite these advances, the landscape of FRLs in cervical cancer—particularly how they intersect with the distinct viral etiology of specific HPV subtypes—remains largely uncharted. Unraveling these specific interactions promises not only to deepen our biological understanding but to unveil novel translational targets for a disease where new therapeutic avenues are urgently needed.

New findings show that a tumor cell’s sensitivity to ferroptosis depends not only on its iron balance and antioxidant capacity, but also on its metabolic profile, particularly how it reprograms its lipid metabolism. Since ferroptosis is triggered by the peroxidation of polyunsaturated fatty acids (PUFAs) in cell membranes, the state of lipid metabolism is now considered a determining factor in sensitivity to ferroptosis (7). In order to reduce lipid peroxidation of membranes and develop tolerance, cancer cells can increase their monounsaturated fatty acid (MUFA) content, stimulate the formation of lipid droplets, or alter fatty acid distribution (19). Mechanistically, tumors often remodel their lipid architecture, specifically shifting fatty acid composition and storage, to construct a metabolic barrier against ferroptotic death. In the landscape of cervical cancer, HR-HPV infection acts as a primary catalyst for this metabolic transformation. While the viral oncoproteins E6 and E7 are notorious for dismantling the p53 and Rb tumor suppressor checkpoints, their influence extends deeply into metabolic regulation, most notably through the hyperactivation of the PI3K/AKT/mTOR axis (20). This signaling surge likely orchestrates a metabolic rewiring that fortifies the cell’s antioxidant capacity, equipping HPV-positive cells with an enhanced ability to buffer the oxidative stress that would otherwise trigger cell death. Such adaptation serves a dual purpose: fueling rapid proliferation while simultaneously shielding the tumor from oxidative damage. Consequently, systematically mapping the intersection of HPV infection, lipid metabolism, and ferroptosis resistance is not merely an academic exercise, but a critical step toward uncovering the metabolic vulnerabilities of these tumors.

To address this challenge, we integrated multi-omics data from TCGA and UCSC Xena to identify FRLs features with strong prognostic capability. From this computational framework emerged LINC02178, a candidate exhibiting a striking correlation with HPV16 infection, a pattern we subsequently validated in clinical specimens. Moving beyond correlation, we utilized *in vitro* models to dissect the functional repertoire of LINC02178, probing its specific contributions to cellular proliferation, migration, and the modulation of ferroptotic phenotypes. Ultimately, this study aims to bridge the gap between risk stratification and molecular mechanism, offering both a predictive tool for clinicians and a clearer understanding of how HPV-driven tumors survive metabolic stress.

## 2 Materials and methods

### 2.1 Acquisition and preprocessing of public database data

To establish a robust foundation for our transcriptomic analysis, we retrieved RNA-sequencing data and corresponding clinical annotations from The Cancer Genome Atlas (TCGA) and the UCSC Xena platform. Focusing specifically on the Cervical Squamous Cell Carcinoma and Endocervical Adenocarcinoma (CESC) cohort, we compiled a total of 319 samples, a dataset dominated by 306 tumor tissues but also including 13 normal cervical controls to serve as a biological baseline. Key clinical variables including patient age, tumor grade, and survival metrics were integrated to support downstream prognostic modeling. Simultaneously, we defined the molecular landscape of ferroptosis by interrogating the FerrDb database; from an initial pool of 844 candidates, we curated a refined list of 564 unique ferroptosis-related genes after rigorously removing redundancies. All raw sequencing counts were subsequently processed in the R environment, where they were standardized to Fragments Per Kilobase of transcript per Million mapped reads (FPKM) and log2-transformed to correct for data skewness and ensure statistical comparability across the cohort. Gene annotation relied on the Ensembl annotation file to distinguish protein-coding genes from lncRNAs, from which the lncRNA expression matrix was extracted for further analysis. Only patient samples with complete RNA sequencing data and corresponding clinical follow-up information were included in this study. Samples with missing vital status or follow-up time, zero survival time, and incomplete clinical data were excluded.

### 2.2 Screening and differential analysis of FRLs

Pearson correlation analysis was employed to assess the correlation in expression between lncRNAs and ferroptosis-related genes. The screening criteria were established as |r| > 0.3 and *P* < 0.05, thereby defining eligible lncRNAs as FRLs subsequently, the “limma” package in R software was utilized to compare the expression differences of FRLs between tumor tissues and normal tissues, with the screening criteria set to |log2 FC| > 1 and *P* < 0.05. The differentially expressed FRLs were visualized through volcano plots and heat maps.

### 2.3 Construction and validation of RSM

Patients meeting the inclusion criteria were randomly divided into a training set and a validation set in a 6:4 ratio. In the training set, FRLs significantly associated with overall survival (OS) (*P* < 0.05) were initially identified through univariate Cox proportional hazards regression analysis. Subsequently, the “glmnet” package was employed for LASSO (Least Absolute Shrinkage and Selection Operator) Cox regression analysis. To strictly prevent model overfitting and select the most robust prognostic features, the optimal penalty parameter λ was determined using 10-fold cross-validation to construct the FRLs RSM. The risk score was calculated using the formula: Risk score = Σ (*β_i_* × Expr*_i_*), where *β_i_* represents the regression coefficient of the corresponding FRL and Expr*_i_* denotes its normalized expression level. Based on the median risk score, patients were classified into high-risk and low-risk groups.

### 2.4 Clinical sample collection and qPCR verification

Clinical cervical swab samples were collected from the Third Affiliated Hospital of Zunyi Medical University (the First People’s Hospital of Zunyi) between November 2024 and July 2025. Patients who underwent HPV testing and had a documented sexual history were required to abstain from sexual activity for 3 days prior to sampling. Additionally, they needed to be in non-menstrual, non-pregnant, and non-lactating states, with no history of anti-HPV treatment, diabetes, or systemic diseases necessitating long-term hormone treatment. Patients were excluded if they had vaginal irrigation, medication, or sexual activity within 3 days before sampling; if they were using contraceptives, antibiotics, or immunosuppressants in that timeframe; or if they had serious medical or surgical conditions, were immunocompromised, or had incomplete clinical data. Based on the results of HPV detection, the samples were categorized into groups of HPV16 single infection, HPV52 single infection, HPV16 multiple infection, HPV52 multiple infection, and other subtypes of multiple infections. Among these, 68 cases were negative samples, 68 cases were single HPV16 infections, 87 cases were single HPV52 infections, 50 cases were multiple HPV16 infections, 96 cases were multiple HPV52 infections, and 48 cases involved multiple infections with other subtypes. At the beginning of our analysis, we extracted all nucleic acid from cervical swab samples using a commercially available extraction kit (Sansure Biotech, China). The resulting product was then transferred directly into qPCR experiments to measure the relative expression levels of important lncRNAs identified in our previous screening. Specific protocols for PCR reaction preparation, amplification, and sequencing are available. All extracted nucleic acid samples were stored at -80 °C for future use.

### 2.5 Cell culture

We first obtained the human cervical cancer cell line SiHa and normal cervical epithelial cells (Hcer Mock) from the cell bank of the Chinese Academy of Sciences. Both cell types were cultured under standard conditions: in glucose-rich DMEM medium enriched with 10% fetal bovine serum and L-glutamine, and kept at 37 °C in a humidified incubator with 5% CO_2_.

### 2.6 Cell transfection and drug treatment

To study the role of LINC02178, we developed a siRNA specific for this lncRNA, as well as a corresponding negative control siRNA. Cell transfection was performed using Lipo8000™ transfection reagent (Beyotime, China) according to the manufacturer’s protocol. Twenty-four hours after transfection, total RNA was extracted and transcribed into cDNA. We then measured the relative expression of LINC02178 by real-time quantitative polymerase chain reaction (RT-qPCR) to confirm the efficacy of the inhibition. Detailed experimental protocols regarding primer sequences, reverse transcription conditions, and amplification settings have been cataloged to ensure reproducibility. Following the targeted knockdown of LINC02178 via siRNA transfection, we sought to determine whether this genetic disruption altered the cells’ sensitivity to metabolic stress. To test this, experimental groups were challenged with either Erastin, a potent ferroptosis inducer, or Ferrostatin-1, a specific inhibitor, while a DMSO-treated cohort served as the solvent baseline. This setup allowed us to isolate the specific contribution of the lncRNA to the ferroptotic process before harvesting the cells for downstream biochemical and functional profiling.

### 2.7 Evaluation of malignant phenotypes: proliferation and migration

To determine if the interplay between LINC02178 silencing and ferroptosis induction compromised the cells’ growth potential, we monitored viability trajectories using a CCK-8 assay. Cells were seeded into 96-well plates with five technical replicates per condition to minimize experimental noise. At designated time points (0, 24, and 48 hours), metabolic activity was quantified by measuring absorbance at 450 nm following a one-hour incubation with the CCK-8 reagent; these readings were then normalized to the 0-hour baseline to accurately visualize dynamic growth trends. Complementing this viability data, we assessed migratory capacity through a wound-healing scratch assay. Once the monolayers reached near-confluence (80–90%), a standardized gap was introduced, and wound closure was tracked photographically at the same intervals. Using ImageJ software to measure the scratch width, we calculated migration as the percentage of wound closure relative to the initial injury, providing a direct readout of the cells’ ability to spread and recover.

### 2.8 Detection of ferroptosis-related biochemical indicators

To further evaluate the induction of ferroptosis following siRNA transfection and pharmacological intervention, we profiled a panel of core intracellular biomarkers. Specifically, we quantified the concentrations of reduced GSH, MDA—a key marker of lipid peroxidation—and intracellular Fe²^+^ using specific assay kits purchased from Beyotime and Applygen (China). Absorbance readings were acquired via a microplate reader, with all data normalized to total protein concentration to ensure comparability, strictly adhering to the manufacturers’ standard protocols. To capture the broader oxidative stress landscape, we assessed intracellular reactive oxygen species (ROS) accumulation using the DCFH-DA fluorescent probe. Post-treatment, cells were harvested and washed with PBS before undergoing incubation with DCFH-DA at 37 °C for 20 minutes in a light-protected environment to prevent probe quenching. Following the removal of excess dye, cellular fluorescence was visualized and captured via microscopy, with mean fluorescence intensity quantified to reflect relative ROS activity.

### 2.9 Statistical analysis

Statistical analyses, model construction, and data visualization were performed using R software (v4.4.2), SPSS (v29.0), ImageJ (v1.47), and GraphPad Prism (v10.1.2). Continuous variables that followed a normal distribution are presented as mean ± standard deviation (Mean ± SD), whereas categorical variables are summarized as frequencies and percentages [n (%)]. Comparisons between two groups were conducted using appropriate statistical tests based on data distribution. For data that did not conform to a normal distribution, nonparametric methods (such as the Mann-Whitney U test) were applied. Comparisons among three or more groups were performed using the Kruskal–Wallis test, followed by Dunn’s *post hoc* multiple comparison test when a significant overall difference was observed. To account for multiple testing in our bioinformatic and clinical expression analyses, *P*-values were adjusted using the Benjamini-Hochberg procedure to control the False Discovery Rate (FDR). All statistical tests were two-sided, and a *P* value < 0.05 (or FDR < 0.05 where applicable) was considered statistically significant.

## 3 Results

### 3.1 Differential expression characteristics of FRLs in cervical cancer

**Figure 1:**
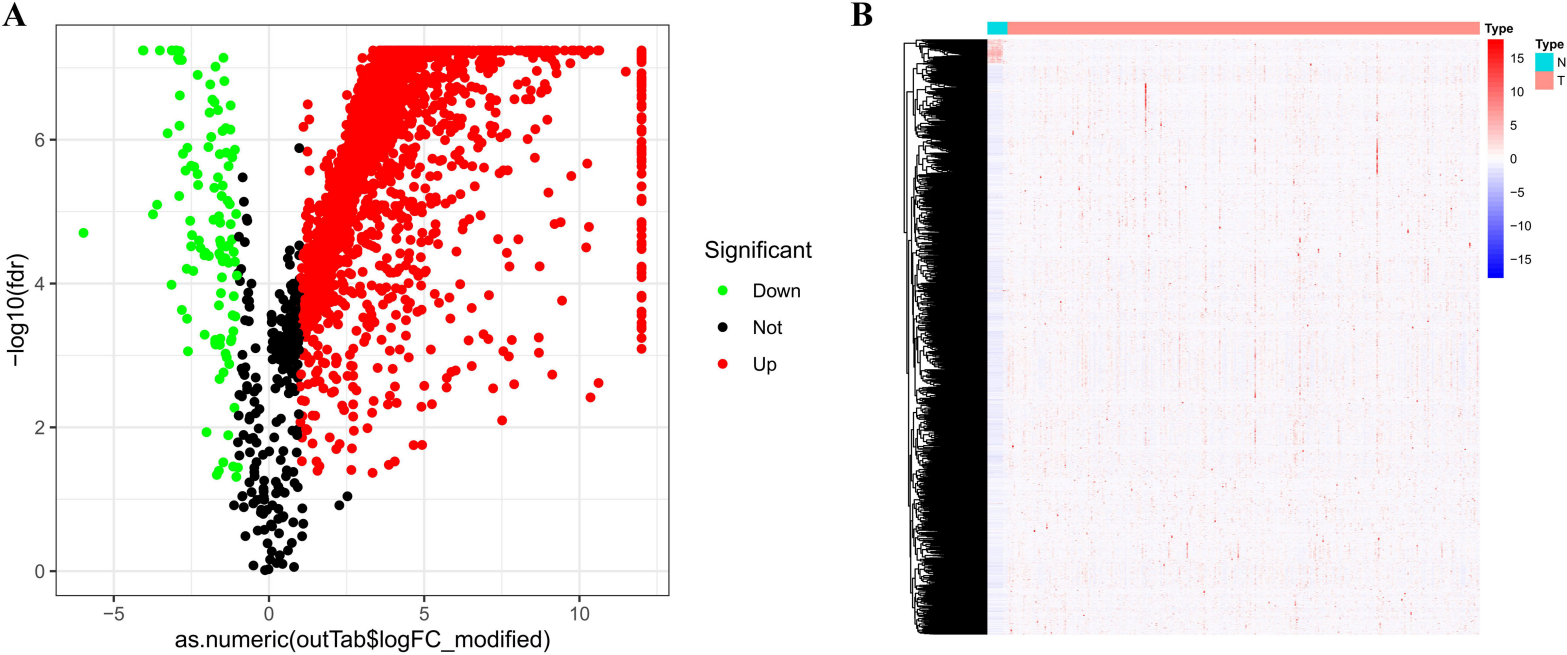
Illustrates the differential expression profile of FRLs in cervical cancer. **(A)** The volcano plot displays the differential expression of FRLs between cervical cancer tissues and normal cervical tissues, derived from the integrated dataset of the TCGA-CESC project. **(B)** The hierarchical clustering heatmap shows differentially expressed FRLs, where red indicates high expression and green indicates low expression. In this heatmap, rows represent genes and columns represent samples.

To systematically map the landscape of FRLs within the context of cervical cancer, we initiated our study by harmonizing patient transcriptomic profiles from the TCGA and UCSC Xena databases, specifically focusing on the CESC cohort. Rather than viewing these transcripts in isolation, we sought to anchor them biologically by interrogating their relationship with the known ferroptosis machinery defined by the FerrDb database. Through a rigorous Pearson correlation analysis—using strict thresholds of |r| > 0.3 and *P* < 0.05 to ensure statistical robustness—we uncovered a dense regulatory network. This computational screening successfully identified 2,747 lncRNAs that exhibited significant co-expression patterns with 461 ferroptosis-related genes, providing a broad candidate pool for subsequent prognostic modeling. Another differential expression analysis (|log_2_FC| > 1, *P* < 0.05) revealed 2,330 FRLs that were deregulated in tumor tissues: 2,140 were overexpressed compared to normal samples and 113 were underexpressed (**Figure 1A**). Hierarchical clustering confirmed that these differentially expressed FRLs had distinct expression profiles, clearly separating cervical cancer tissues from normal cervical tissues at the transcriptional level (**Figure 1B**).

### 3.2 RSM Construction process based on FRLs

**Figure 2:**
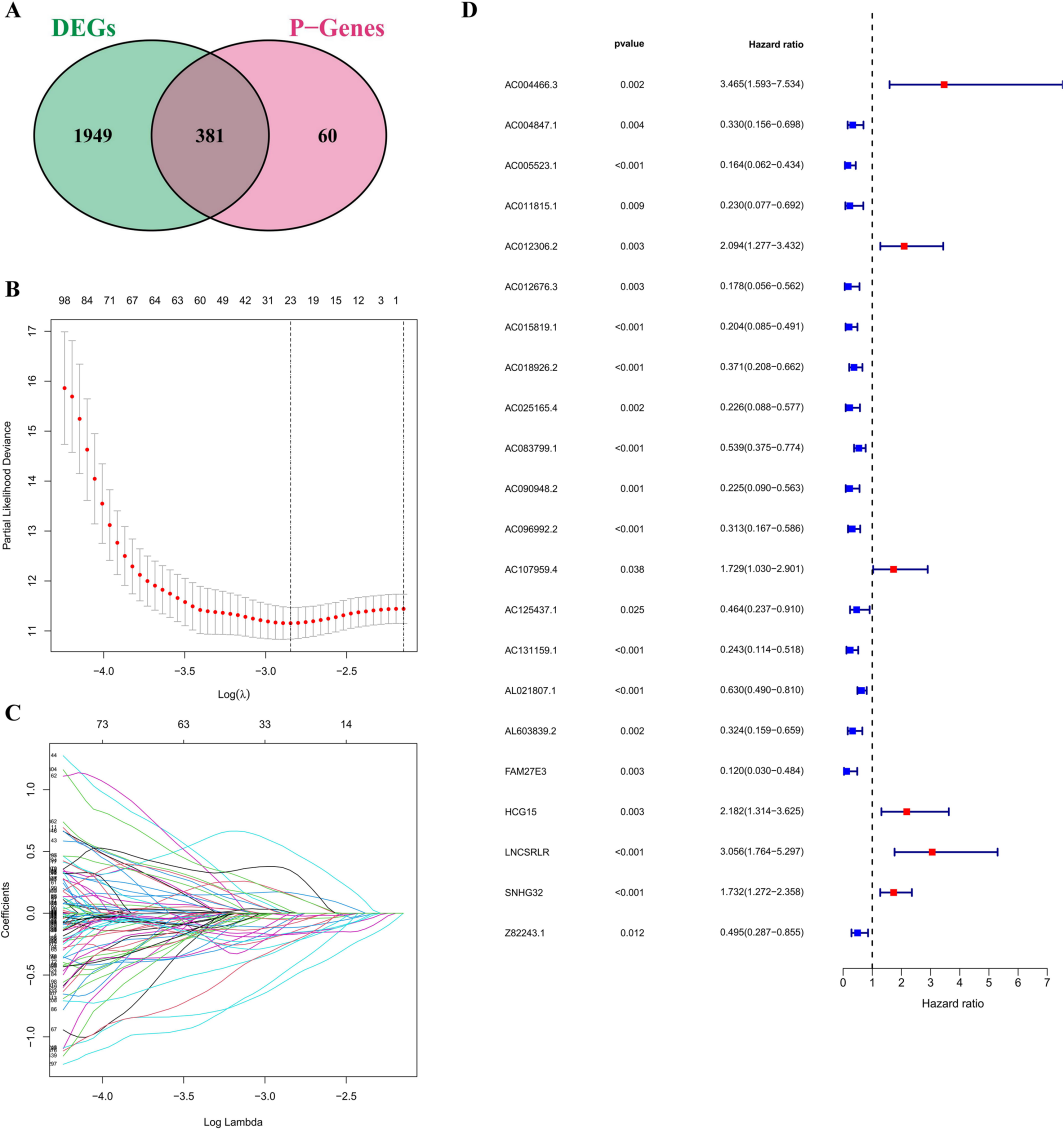
Shows the process of creating the FRLs-based RSM in cervical cancer. **(A)** Cross-sectional analysis of differentially expressed FRLs and prognostic FRLs. **(B)** Evolution of coefficient changes for FRLs during LASSO regression. **(C)** Selection of the optimal λ from tenfold cross-validation error curves. **(D)** Forest plot with the 22 FRLs included in the final risk model.

To evaluate the potential value of FRLs in predicting the prognosis of cervical cancer patients, a ferroptosis-related gene expression matrix was obtained and the patients were randomly divided into a training set and a validation set at a ratio of 6:4. The expression matrix of FRLs was integrated with the corresponding clinical survival information. Univariate Cox proportional hazards regression analysis was performed using the ‘survival’ package in R software on the training set to systematically evaluate the association between FRLs and OS. Under the significance threshold (*P* < 0.05), 441 FRLs were found to be significantly associated with OS, of which 67 were identified as having a hazard ratio (HR > 1), indicating that their high expression was significantly related to poor prognosis. Conversely, 374 FRLs were identified as protective factors (HR < 1), with high expression associated with better clinical outcomes. Based on Cox univariate analysis, we then performed an intersection analysis, combining differentially expressed FRLs with those associated with prognosis. This step identified 381 candidate FRLs that were both differentially expressed and significantly associated with patient outcomes (**Figure 2A**). To reduce overfitting and collinearity, we applied LASSO Cox regression to these candidates and adjusted the penalty parameter λ by tenfold cross-validation (**Figures 2B, C**). From this, we created a signature of 22 FRLs that forms the core of our risk assessment model (**Figure 2D**). This multigene signature captures several transcription patterns related to ferroptosis and is a useful tool for patient risk stratification and as a guide for further functional studies.

### 3.3 Evaluation of the predictive performance of RSM in cervical cancer

**Figure 3:**
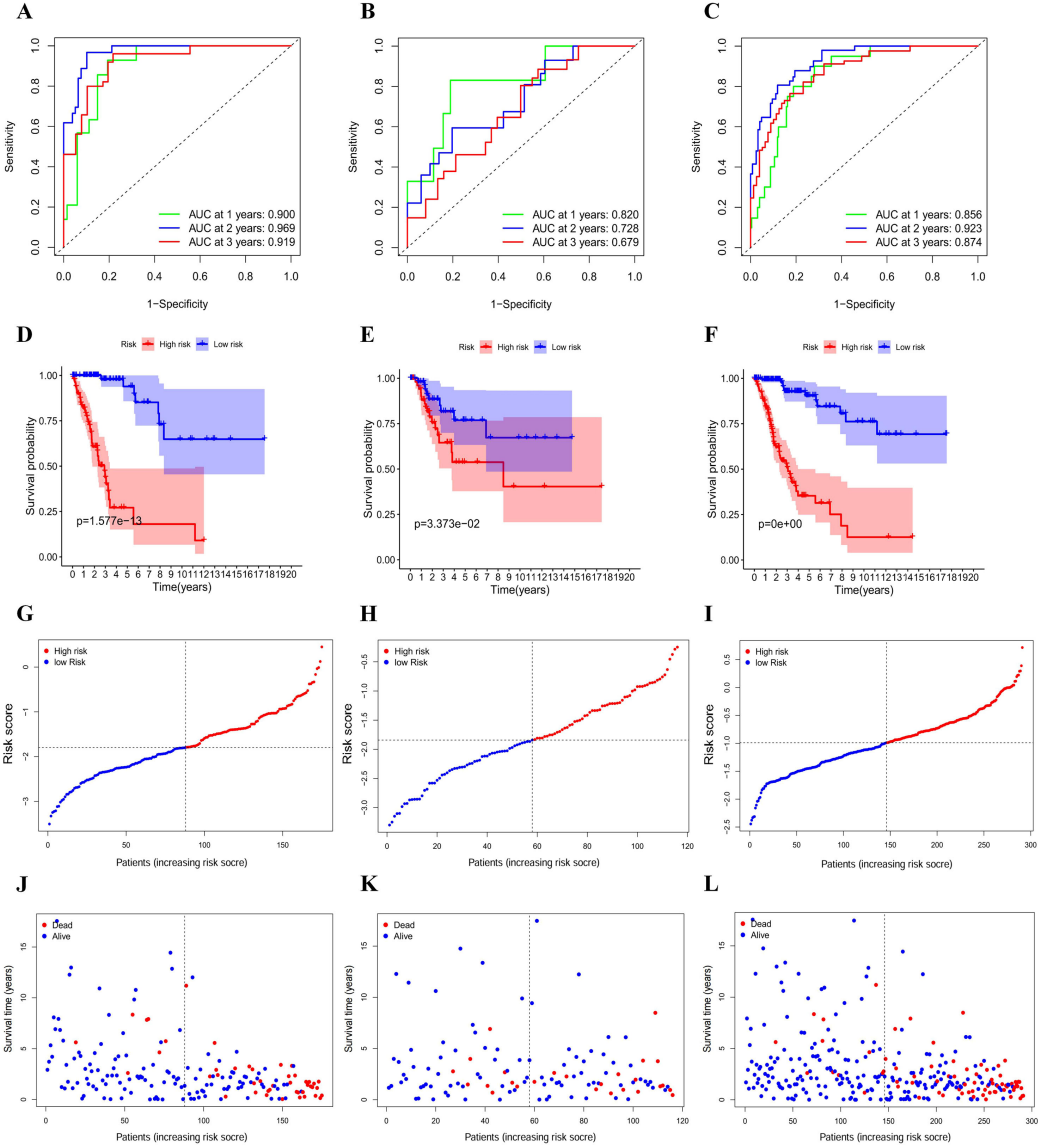
Evaluation of the RSM in cervical cancer. **(A–C)** Time-dependent ROC curves for the training, validation, and total cohorts. **(D–F)** Kaplan-Meier OS curves, stratified according to RSM grouping. **(G–I)** Distribution of risk scores across cohorts. **(J–L)** Survival status of patients according to risk scores.

Using the regression coefficients from the LASSO-Cox analysis, we calculated a risk score for each patient in both the training cohort and the validation cohort. Patients were then divided into high-risk and low-risk groups based on the median risk score from the training set. Time-dependent ROC analysis showed that the model had strong predictive power over different follow-up periods. In the training set, the area under the curve (AUC) values for OS at 1, 2, and 3 years were 0.900, 0.969, and 0.919, respectively. The corresponding values in the validation set were 0.820, 0.728, and 0.679, with stable performance observed in the combined cohort (**Figures 3A–C**).

Kaplan-Meier analysis confirmed that high-risk patients had significantly shorter OS than low-risk patients, a trend that was maintained in the training set, validation set, and overall cohort (P < 0.001) (**Figures 3D–F**). Further analysis of the distributions of risk scores and patient survival status showed that fatal events increased with higher risk scores and that the proportion of deaths was significantly higher in the high-risk group (**Figures 3G–L**). Overall, these results confirm the model’s ability to effectively stratify prognostic risk in patients with cervical cancer.

### 3.4 Independent prognostic value and clinical correlation analysis of RSM in cervical cancer

**Figure 4:**
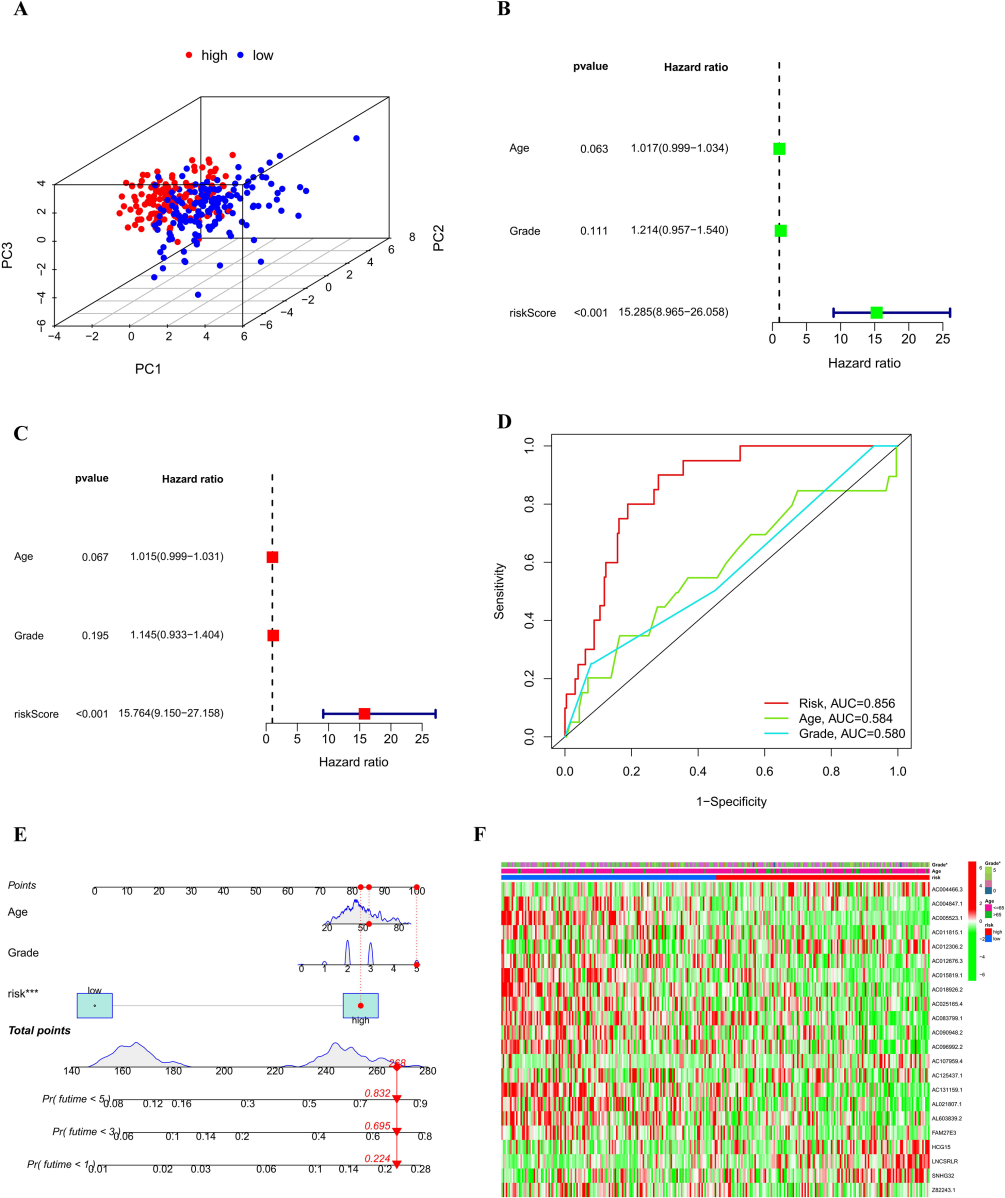
Illustrates the independent prognostic value and clinical relevance of RSM in cervical cancer. **(A)** PCA comparing patients in the high-risk group to those in the low-risk group. **(B)** Forest plot depicting the results of univariate Cox proportional hazards regression analysis. **(C)** Forest plot presenting the findings of multivariate Cox proportional hazards regression analysis. **(D)** ROC curve comparison of RSM and clinical factors for predicting prognosis. **(E)** Nomogram prediction model along with the calibration curve that integrates risk score and clinical factors. **(F)** Correlation analysis between RSM and clinicopathological features. *P < 0.05.

To further evaluate whether the RSM provides independent prognostic value, we first performed a principal component analysis (PCA) of the global transcriptomes of patients classified into high- and low-risk groups. PCA revealed a distinct segregation between the high and low-risk groups within the expression profile space, suggesting that the risk score captures fundamental differences in the underlying molecular landscape (**Figure 4A**). To determine whether this signature could serve as a standalone prognostic tool, we subjected the risk score to both univariate and multivariate Cox regression analyses. In the univariate model, the risk score exhibited a potent association with OS (HR = 15.285, 95% CI: 8.965–26.058, *P* < 0.001). Crucially, this predictive capacity remained robust even after adjusting for potential confounders such as age and tumor grade in the multivariate analysis, confirming the risk score as an independent predictor of patient outcome (HR = 15.764, 95% CI: 9.150–27.158, *P* < 0.001) (**Figures 4B, C**). The superiority of the RSM was further highlighted by ROC analysis, where it achieved an AUC of 0.856, markedly outperforming traditional clinical metrics like age (AUC = 0.584) and tumor grade (AUC = 0.580) (**Figure 4D**).

Building on these findings, we constructed a nomogram integrating the risk score with key clinical variables to provide a practical tool for estimating 1, 3, and 5-year survival probabilities (**Figure 4E**). Calibration curves showed good agreement between the nomogram’s predictions and the actual observed results, confirming its clinical applicability. Furthermore, correlation analysis between risk groups and clinicopathological features revealed that patients in the high-risk group were significantly associated with higher tumor grades (*P* < 0.05) (**Figure 4F**), suggesting a correlation between risk scores and disease progression characteristics.

### 3.5 Screening of candidate FRLs based on RSM and their expression characteristics related to HPV infection

Following the construction of the FRLs RSM and the implementation of patient risk stratification, we aimed to further identify FRL candidates of clinical validation value from the model-related molecular background. Patients in the TCGA-CESC cohort were categorized into high-risk and low-risk groups based on the median risk score calculated by the RSM. The expression differences of FRLs between these two groups were compared within the framework of this model-driven risk stratification, leading to the identification of three key FRLs (FDR < 0.05) as outlined in **Table 1**.

**Table 1:** Expression characteristics of key FRLs.

Gene	Low mean	High mean	logFC	*P-*value	fdr
LINC02178	1.649319863	8.204244828	2.314499328	5.48E-03	1.43E-02
AL139246.3	2.125171918	0.496398621	-2.098008542	1.57E-08	6.39E-07
AL157931.1	5.676367808	0.800871034	-2.825326227	2.87E-03	8.46E-03

**Figure 5:**
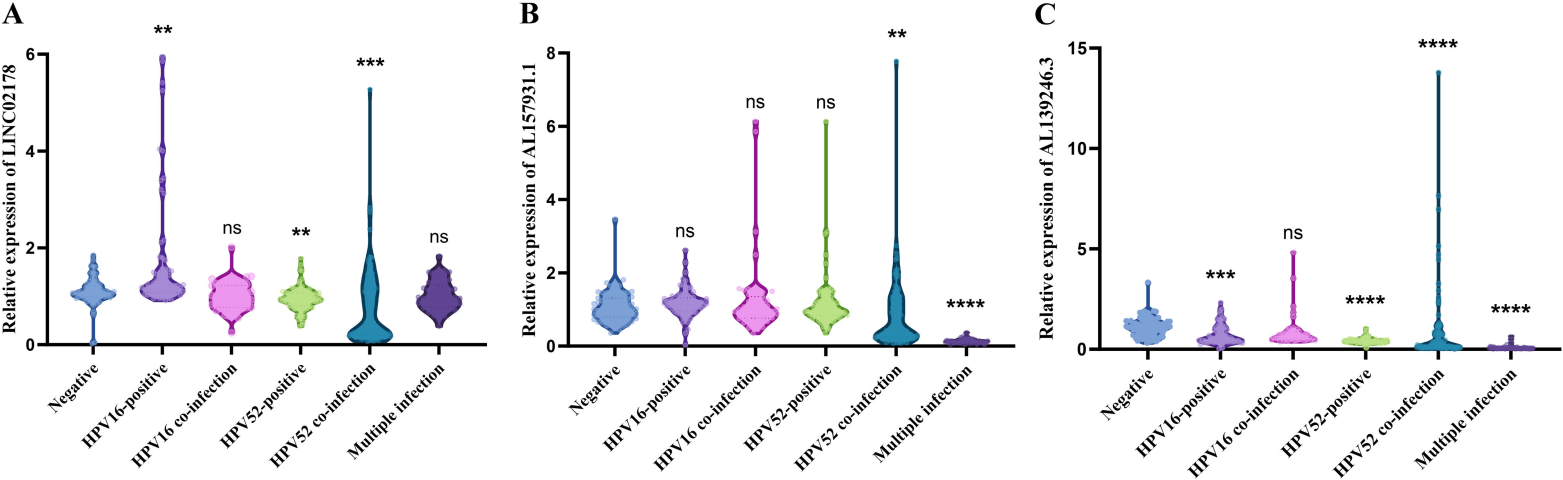
Expression patterns of FRLs important for HPV infection subtypes in clinical samples. **(A)** Relative expression of LINC02178. **(B)** Relative expression of AL157931.1. **(C)** Relative expression of AL139246.3. Significance markers: ns (not significant, *P* > 0.05), **P < 0.01, ***P < 0.001, ****P < 0.0001. All comparisons were adjusted using the false discovery rate (FDR < 0.05).

To validate our bioinformatics findings in a real-world clinical setting, we analyzed cervical swab samples from a precancerous and early screening cohort. The detailed baseline demographic and clinical characteristics of this cohort (N = 417), including age and specific HPV infection status, are summarized in **Table 2**. We evaluated the expression levels of three ferroptosis-related lncRNAs—LINC02178, AL157931.1, and AL139246.3—comparing them against a backdrop of HPV-negative cases versus various HPV infection subtypes. Overall, all three lncRNAs showed significant variation across the groups (*P* < 0.0001), but the way they behaved was distinct for each gene. For instance, LINC02178 exhibited distinct expression patterns dependent on the specific HPV infection status. Compared to the HPV-negative group, levels spiked significantly in samples with a single HPV16 infection. Conversely, in both single and multiple HPV52 infections, LINC02178 expression was significantly downregulated. Interestingly, no significant differences were observed in the HPV16 multiple infection or other multiple infection groups compared to the negative control. AL157931.1 exhibited a different expression profile. Its levels remained relatively stable in HPV16 single, HPV16 coinfection, and HPV52 single infections, showing no significant change from the negative group. Conversely, in HPV52 coinfections, expression was significantly downregulated, and it trended down even more noticeably in multiple infection samples. Furthermore, AL139246.3 expression was broadly suppressed across most infection subtypes. Compared to the negative group, it was significantly down in HPV16 single, HPV52 single, HPV52 coinfection, and multiple infection samples. The HPV16 coinfection group showed a downward trend too, though it didn’t quite hit statistical significance (**Figure 5**).

**Table 2:** Baseline demographic and clinical characteristics of the screening cohort.

Characteristics	Negative	HPV16 single	HPV52 single	HPV16 co-inf	HPV52 co-inf	Other mult	Total
	n=68	n=68	n=87	n=50	n=96	n=48	N=417
Age (years), n (%)							
≤30	15 (22.1)	9 (13.2)	13 (14.9)	7 (14.0)	11 (11.5)	6 (12.5)	61 (14.6)
31-45	30 (44.1)	28 (41.2)	42 (48.3)	16 (32.0)	37 (38.5)	19 (39.6)	172 (41.2)
46-60	20 (29.4)	23 (33.8)	29 (33.3)	15 (30.0)	35 (36.5)	22 (45.8)	144 (34.5)
>60	3 (4.4)	8 (11.8)	3 (3.4)	12 (24.0)	13 (13.5)	1 (2.1)	40 (9.6)

All in all, these three ferroptosis-related lncRNAs show distinct expression fingerprints depending on the HPV subtype, pointing to a tight link between HPV infection status and how these transcripts get regulated.

### 3.6 Study of the link between LINC02178 and cellular ferroptosis status

To dig deeper into how these three FRLs might actually be “pulling the strings” within the ferroptosis regulatory network, we systematically analyzed their expression correlations with known ferroptosis-related genes using TCGA-CESC data. The results were telling. While all three lncRNAs are clearly plugged into the network, their connection strengths vary in **Table 3**. LINC02178 really stood out, showing a robust positive correlation with PLIN2 (Cor = 0.68, *P* = 7.95E-43), a key player in lipid metabolism. The others showed significant links too—AL139246.3 with PTPN18 (Cor = 0.52, *P* = 2.27E-22) and AL157931.1 with the iron transporter SLC40A1 (Cor = 0.43, *P* = 4.37E-15)—but they didn’t quite match the intensity we saw with LINC02178 (**Table 2**). Looking at the big picture—combining these correlation stats with the unique clinical expression patterns we validated earlier—LINC02178 emerged as the most promising candidate. Consequently, we decided to zero in on LINC02178 for our subsequent experiments. Our next step was to test how this specific lncRNA responds to ferroptosis-targeting drugs and how it influences the biological behavior of cervical cancer cells.

**Table 3:** Correlation analysis between key FRLs and ferroptosis genes.

FerrGene	LncRNA	Cor	*P-*value	Regulation
PLIN2	LINC02178	0.679732492	7.95E-43	postive
PTPN18	AL139246.3	0.517599516	2.27E-22	postive
SLC40A1	AL157931.1	0.428353566	4.37E-15	postive

**Figure 6:**
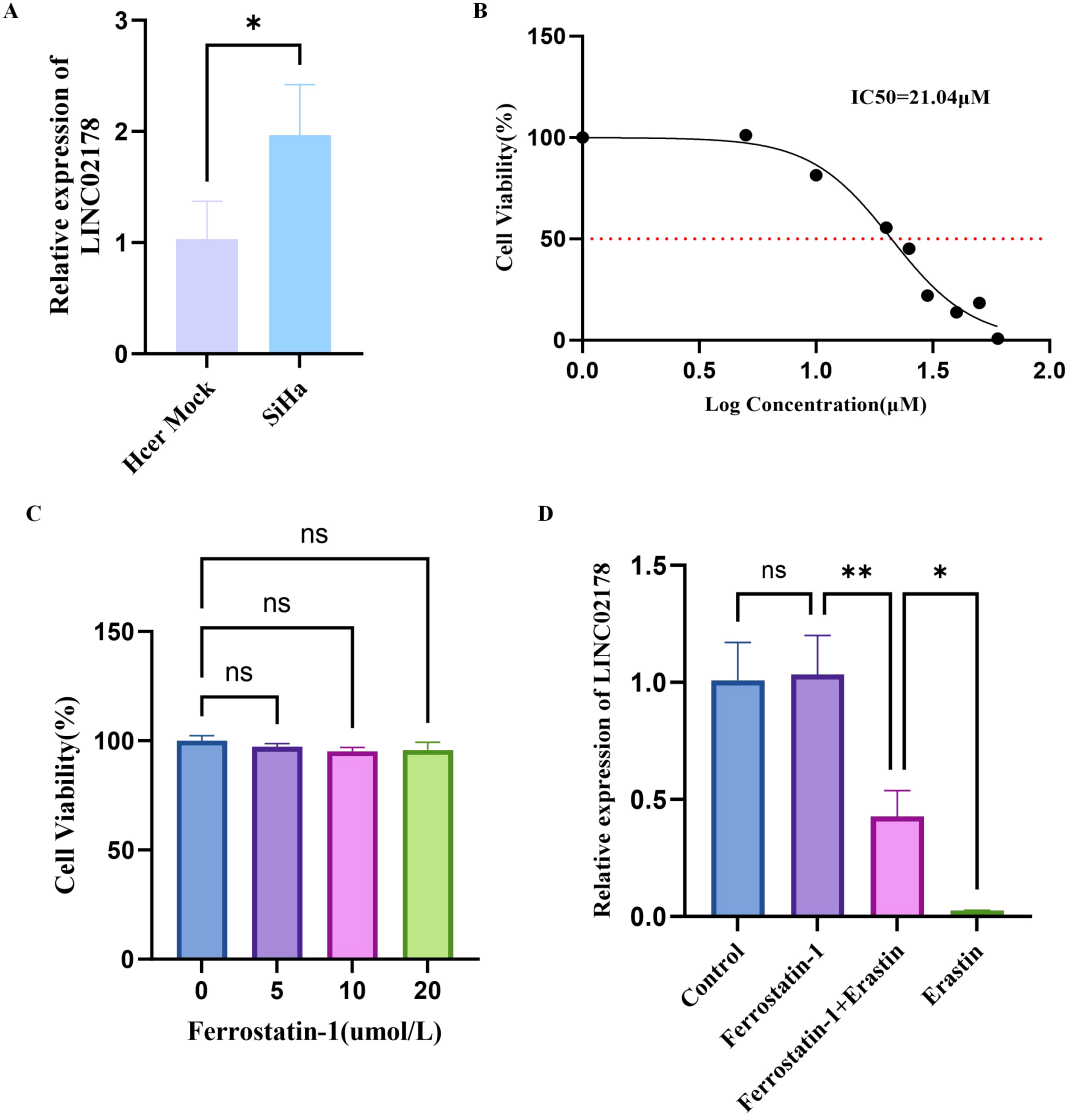
Relationship between LINC02178 and ferroptosis status in HPV16-positive SiHa cells. **(A)** Expression of LINC02178, detected by RT-qPCR. **(B)** Dose-response relationship and IC_50_ of Erastin. **(C)** Effect of Ferrostatin-1 on cell viability. **(D)** Changes in LINC02178 expression under ferroptosis-modulating treatments. Significance markers: ns (not significant, *P* > 0.05), *P < 0.05, **P < 0.01.

In HPV16-positive SiHa cervical cells, RT-qPCR confirmed that LINC02178 expression was significantly higher than in control cells (**Figure 6A**), consistent with its upregulation in HPV16-positive clinical samples. To investigate the link between LINC02178 and ferroptosis, we treated SiHa cells with the ferroptosis inducer Erastin and the inhibitor Ferrostatin-1. CCK-8 assays showed that Erastin suppressed growth in a dose-dependent manner, with an IC_50_ of 21.04 μM (**Figure 6B**), while Ferrostatin-1 alone (1-20 μM) had no significant effect on viability (**Figure 6C**). Under these conditions, we again measured LINC02178 expression. Treatment with Erastin significantly reduced LINC02178 levels compared to the DMSO control, while Ferrostatin-1 alone had only a minor effect. Interestingly, pretreatment with Ferrostatin-1 partially counteracted the Erastin-induced downregulation of LINC02178 (**Figure 6D**).

### 3.7 Effect of LINC02178 knockdown on the biological behavior of HPV16-positive cervical cancer cells

**Figure 7:**
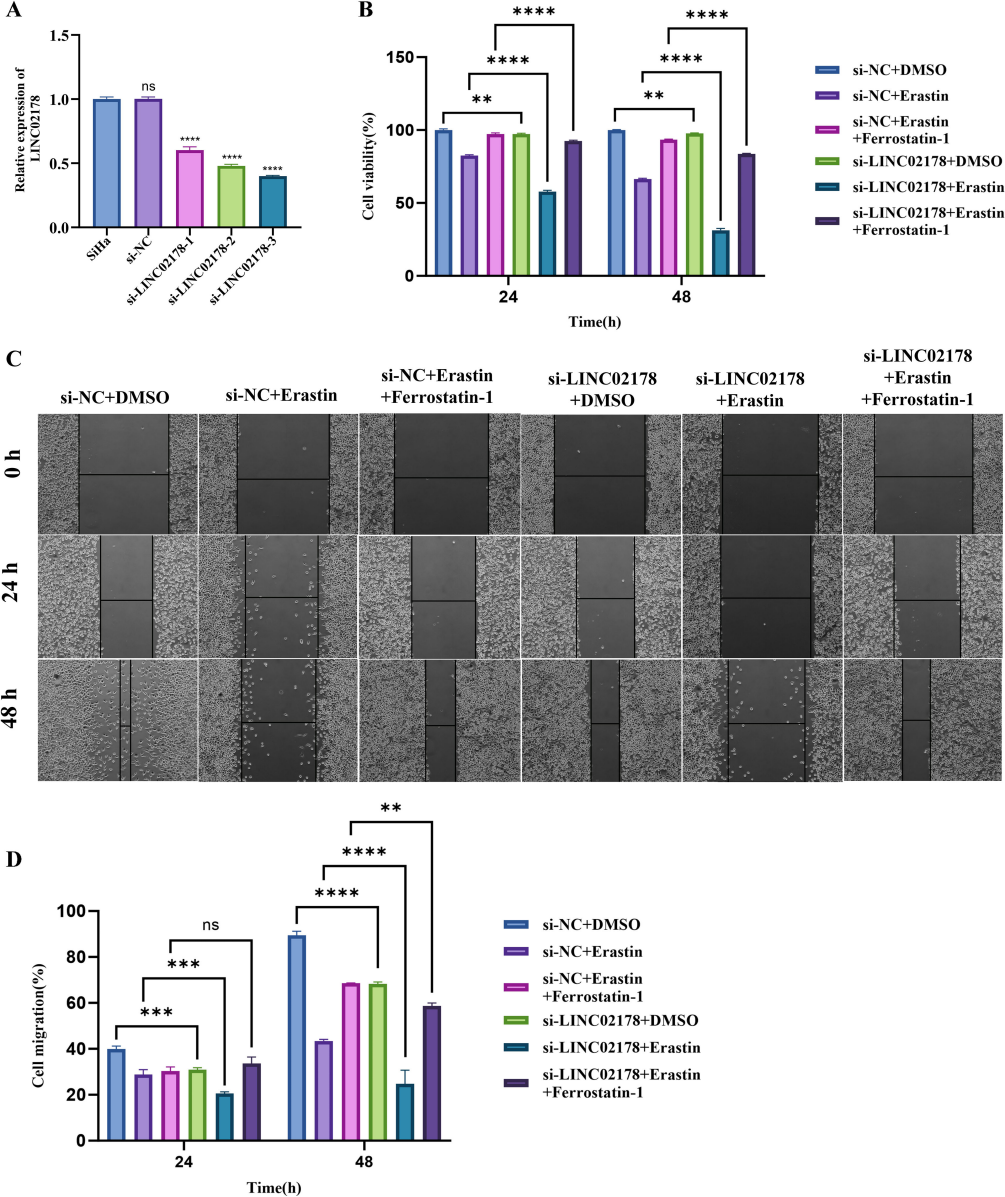
Functional impact of LINC02178 inhibition on SiHa cell proliferation and migration. **(A)** Verification of knockdown efficiency via RT-qPCR following siRNA transfection. **(B)** CCK-8 viability curves illustrating the suppressive effects of indicated treatments on cell growth over time. **(C)** Representative photomicrographs from the scratch wound healing assay. **(D)** Quantitative analysis of migration rates, highlighting the synergistic inhibition driven by Erastin and LINC02178 silencing and its partial rescue by Ferrostatin-1. Statistical significance was determined as indicated. ns, not significant; **P < 0.01; ***P < 0.001; ****P < 0.0001.

We began by studying three siRNAs targeting LINC02178 in HPV16-positive SiHa cells. RT-qPCR confirmed that all three significantly reduced LINC02178 expression compared to the si-NC control. Since si-LINC02178–3 had the most potent inhibitory effect, we selected it for all subsequent experiments (**Figure 7A**). Using the CCK-8 assay, we found that Erastin alone significantly inhibited SiHa cell proliferation compared to the si-NC+DMSO group. Inhibition of LINC02178 also reduced proliferation. In combination with LINC02178 inhibition, the antiproliferative effect of Erastin was further enhanced. Addition of the ferroptosis inhibitor Ferrostatin-1 partially, but not completely, reversed this inhibition (**Figure 7B**). The scratch wound healing assay revealed a consistent pattern: both Erastin and LINC02178 inhibition significantly slowed cell migration. Mirroring the proliferation results, the scratch wound healing assay revealed that combining LINC02178 knockdown with Erastin precipitated a severe defect in cell motility—an impairment that was notably, though not fully, reversed by the administration of Ferrostatin-1 (**Figures 7C, D**).

### 3.8 Effects of LINC02178 knockdown on ferroptosis-related biochemical indicators

**Figure 8:**
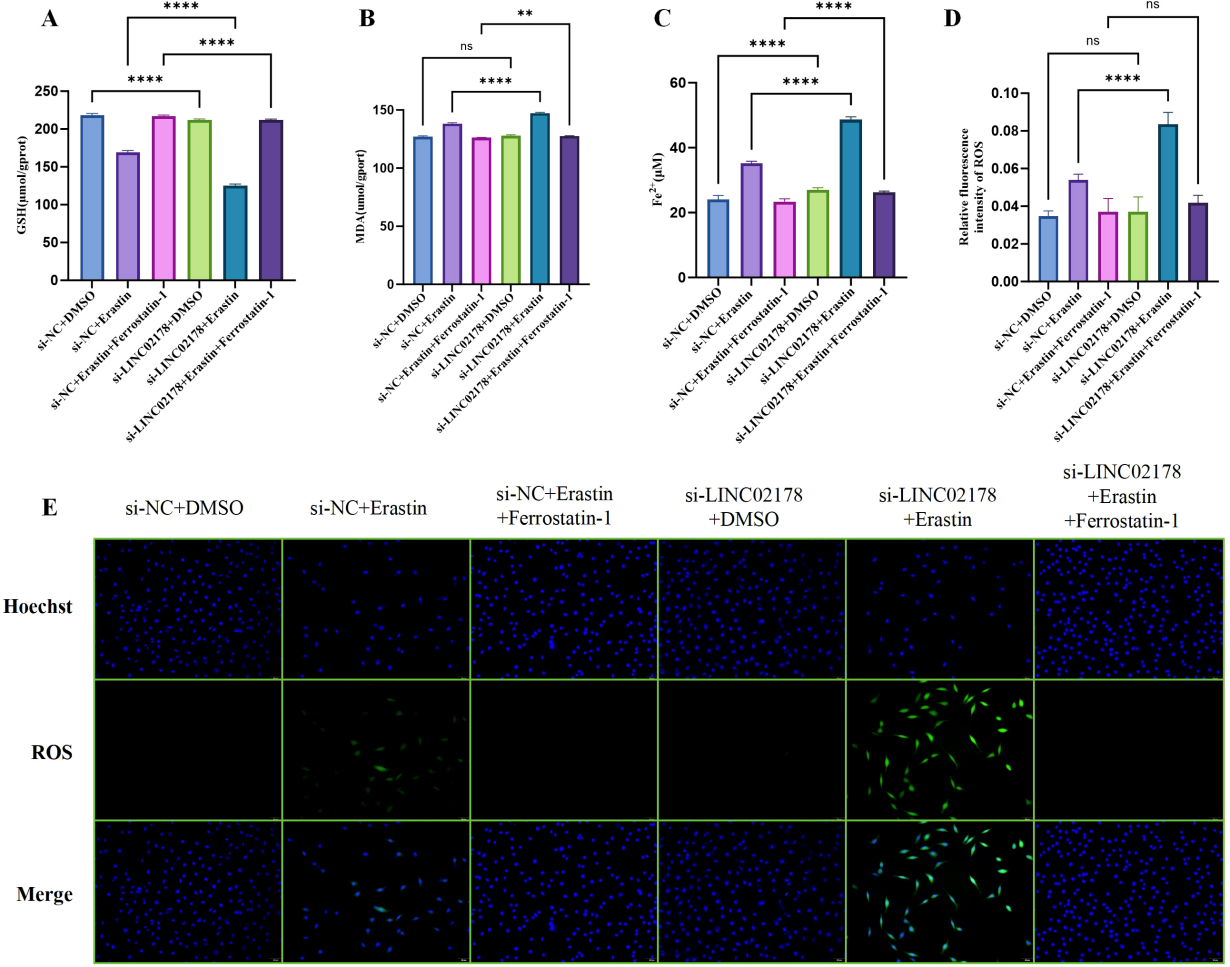
Effect of LINC02178 inhibition on biochemical markers of ferroptosis in SiHa cells. **(A)** Intracellular GSH levels under different treatments. **(B)** Changes in MDA content. **(C)** Changes in intracellular Fe²^+^ content. **(D)** Quantitative analysis of intracellular ROS levels in cells treated with DMSO, Erastin, and Ferrostatin-1 following LINC02178 knockdown. **(E)** Representative fluorescence images showing intracellular ROS accumulation detected by ROS-sensitive fluorescent probe. Statistical significance was determined as indicated. ns, not significant; **P < 0.01; ****P < 0.0001.

To substantiate the phenotypic changes observed in cell viability and migration, we investigated the underlying biochemical footprint of ferroptosis in SiHa cells, focusing on the interplay between LINC02178 status and oxidative defense. By quantifying the intracellular concentrations of reduced GSH, MDA, and Fe²^+^, we aimed to determine if LINC02178 knockdown actively dismantles the cell’s resistance mechanisms. As anticipated, Erastin treatment known to inhibit the cystine/glutamate antiporter, precipitated a decline in GSH levels relative to the si-NC + DMSO control. Crucially, however, this antioxidant depletion was significantly compounded in the si-LINC02178 + Erastin group, indicating that the loss of this lncRNA compromises the cell’s ability to maintain the glutathione reservoir necessary to buffer against lethal lipid peroxidation. Interestingly, LINC02178 inactivation alone also reduced GSH, while the addition of the Ferrostatin-1 inhibitor had only a minor effect on GSH levels (**Figure 8A**). When examining markers of oxidative damage, MDA and Fe²^+^ levels increased significantly after Erastin treatment, with the largest increase observed in the si-LINC02178 + Erastin group. Deactivation of LINC02178 alone also led to an increase in the Fe²^+^, although treatment with Ferrostatin-1 partially counteracted these changes (**Figures 8B, C**). In order to make lipid peroxidation directly visible, we used a fluorescent probe. The signal was strongly amplified by Erastin and became even more evident in combination with LINC02178 inhibition. In contrast, Ferrostatin-1 prevented a significant increase in fluorescence (**Figures 8D, E**).

## 4 Discussion

Cervical cancer remains the fourth most common cancer among women worldwide. Although HPV vaccination and screening have helped reduce overall incidence, outcomes remain poor in advanced or recurrent cases (2, 21). In recent years, growing understanding of tumor metabolic reprogramming and non-classical cell death pathways has highlighted ferroptosis, a form of iron-dependent regulated cell death triggered by lipid peroxides, which appears to play a role in both cancer development and treatment resistance (22–24). However, the role of lncRNAs in regulating ferroptosis in HPV-related cervical cancer has not yet been sufficiently studied.

In this work, we integrated transcriptomic data from TCGA and UCSC Xena to create a risk assessment model for FRLs. Clinical samples and *in vitro* assays were then used to study the function of the key molecules identified. The final model included 22 FRLs and showed strong prognostic performance in the training and validation cohorts, independent of usual clinical factors such as age or tumor grade. It is noteworthy that high-risk patients not only had shorter OS but also tended to have more advanced disease. This observation suggests that ferroptosis-related genetic signatures are not merely passive byproducts of cellular stress but active participants in tumor expansion, mirroring aggressive patterns recently documented in esophageal and liver cancers (25, 26). Guided by our clinical screening, we observed distinct expression fingerprints for the three top FRL candidates—LINC02178, AL157931.1, and AL139246.3—across different HPV subtypes. While AL139246.3 and AL157931.1 exhibited broader suppression across various infection subtypes or multiple infection scenarios, LINC02178 emerged as a uniquely compelling target due to its significant upregulation specifically in HPV16 single-infection swab samples. Consequently, we zeroed in on LINC02178 for downstream functional investigation. It is noteworthy, however, that while LINC02178 is upregulated in HPV16 single infections, our clinical swab data revealed its significant downregulation in both single and multiple HPV52 infections. Rather than a discrepancy, this divergence intriguingly suggests that metabolic reprogramming during early cervical lesions may be highly subtype-specific. Since HPV16 E6/E7 oncoproteins are known to profoundly remodel cellular metabolism—specifically by enhancing fatty acid and glutamine utilization—it is plausible that HPV16 preferentially exploits the LINC02178 network to evade ferroptosis, whereas HPV52 might rely on alternative adaptive mechanisms or confer a different oxidative stress profile (4, 27, 28). We propose that LINC02178 may serve as a key, HPV16-related metabolic adaptor, potentially enabling infected cells to cultivate a ferroptosis-tolerant phenotype that buffers against oxidative stress while simultaneously securing their survival and migratory potential.

Further mechanistic interrogation revealed a robust positive correlation between LINC02178 and PLIN2 (Cor = 0.68), a canonical regulator of lipid droplet stability known to orchestrate lipid storage and metabolism (19). This association is particularly salient given recent evidence that sequestering polyunsaturated fatty acids within lipid droplets creates a metabolic reservoir that effectively fortifies cancer cells against ferroptotic execution (29, 30). Our functional assays substantiated this link: LINC02178 deactivation precipitated a collapse in antioxidant defense—marked by profound GSH depletion and surging intracellular Fe²^+^, MDA, and ROS levels—signaling a significant metabolic shift toward ferroptotic susceptibility. Importantly, LINC02178 knockdown significantly increased sensitivity to Erastin. These data indicate that LINC02178 plays an important role in maintaining redox homeostasis and protecting cervical cancer cells from excessive oxidative stress. This protective function mirrors its established oncogenic profile in lung adenocarcinoma, where elevated LINC02178 fuels proliferation and averts apoptosis (31); indeed, recent work by Zeng et al. confirmed that targeted inhibition of LINC02178 effectively curbs tumor growth both *in vitro* and *in vivo* (32).

**Figure 9:**
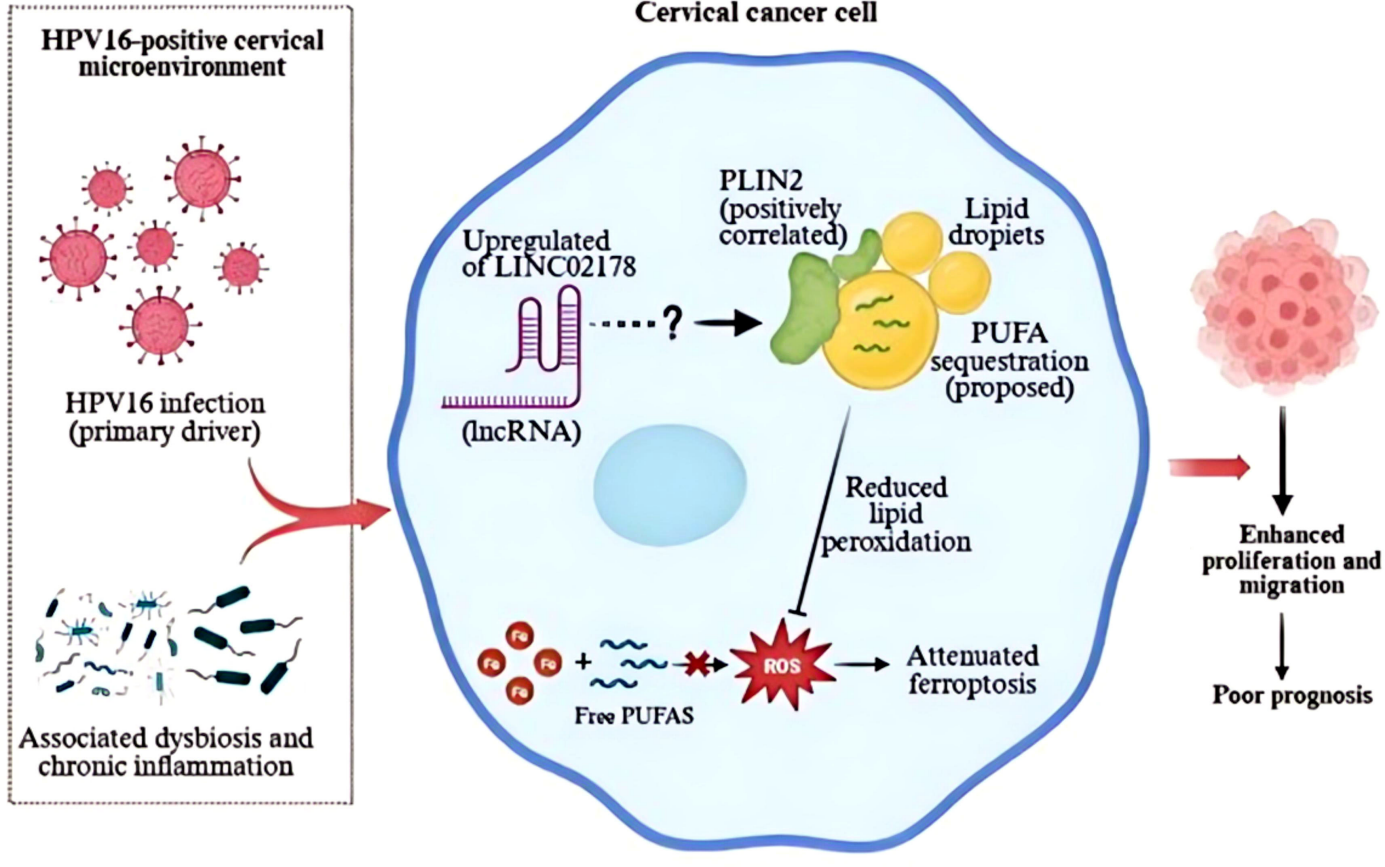
Schematic illustration of the mechanism.

Synthesizing our bioinformatic findings with clinical and functional evidence, we propose a conceptual framework (**Figure 9**) that positions LINC02178 as a critical mediator of ferroptosis resistance in HPV16-positive cervical cancer.

Our data suggest that the virus may strategically exploit the highly co-expressed LINC02178/PLIN2 network to arm infected cells against ferroptotic death, thereby facilitating the progression of high-grade lesions. Intriguingly, this regulation appears to be reciprocal; we observed that LINC02178 expression is not static but actively modulated by the cell’s redox status, pointing to a sophisticated adaptive mechanism. In ferroptosis induction and inhibition experiments, LINC02178 levels decreased significantly after treatment with the ferroptosis inducer Erastin, an effect that was partially reversed by the addition of the inhibitor Ferrostatin-1. This suggests that LINC02178 is sensitive to intracellular oxidative stress. Under normal conditions, higher expression of LINC02178 may help maintain an anti-ferroptosis defense. However, when Erastin depletes GSH and causes significant oxidative stress, this protective feedback appears to break down: LINC02178 expression decreases significantly, pushing cells toward ferroptosis. This dynamic regulation is consistent with previous reports showing that some tumors under high ferroptotic pressure downregulate oncogenic molecules in response to death signals (33). For example, prolonged ferroptotic stress in high-risk precancerous lesions infected with HPV can reduce the expression of the KRAS oncogene; only later, when the lesions progress to cancer, do cells increase KRAS again by strengthening anti-ferroptotic signaling pathways (34). Consequently, the downregulation of LINC02178 in our system could be a sign of a “failure of self-protection mechanisms” under extreme oxidative stress, highlighting its role as an important survival factor for cervical cancer cells.

However, several limitations of the present study must be carefully acknowledged. Firstly, the initial differential expression analysis was constrained by the inherent sample imbalance in the TCGA-CESC cohort (306 tumors vs. 13 normal tissues), and the lack of comprehensive external public datasets restricted our ability to perform independent cohort validation. Nevertheless, we mitigated this bias by strictly relying on the robust survival data of the tumor cohort for our core prognostic modeling. Furthermore, to minimize the risk of overfitting, we employed a stringent 6:4 internal train-validation split, LASSO regression with L1 penalization, and 10-fold cross-validation, which collectively ensured the stability and reliability of the established 22-FRL risk signature. Secondly, we utilized non-invasive cervical swabs from an early screening cohort (including non-cancer and precancerous lesions) rather than deep tissue biopsies from advanced cervical cancer patients. While swabs cannot fully reflect the complex deep microenvironment of invasive solid tumors, the abnormal expression of LINC02178 in these samples intriguingly suggests that ferroptosis resistance may emerge as an early metabolic adaptation during HPV16-driven lesion progression, highlighting its strong translational potential for non-invasive early warning. Thirdly, guided by our clinical findings that LINC02178 upregulation is highly specific to HPV16 infection, our functional validation was deliberately restricted to an HPV16-positive cervical cancer cell line (SiHa) to provide a preliminary *in vitro* proof-of-concept. While this ensures alignment with our clinical data, incorporating diverse HPV-negative models, utilizing lentiviral vectors for stable expression, and conducting *in vivo* animal experiments are essential for future research to verify its broader biological relevance. Fourthly, our mechanistic exploration relied primarily on functional biochemical readouts of ferroptosis (e.g., GSH, MDA, ROS, Fe²^+^). The exact direct regulatory mechanisms (e.g., whether LINC02178 directly interacts with PLIN2 via ceRNA networks) and protein-level expression of core ferroptotic regulators (such as GPX4) require further extensive investigation using direct interaction assays (like RNA pull-down) and Western blotting. Finally, the validation of our clinical samples was based on retrospective cases, and the sample size was relatively limited, analyzing primarily infections of HPV16 and HPV52. To translate these findings into clinical practice, prospective, large-scale studies are essential to validate the applicability of our model and LINC02178 across diverse patient populations. Beyond prognostication, the true translational potential lies in therapeutic intervention; specifically, targeting LINC02178—perhaps via antisense oligonucleotides or siRNA—alongside conventional chemotherapy or ferroptosis inducers warrants rigorous investigation. Such combinatorial strategies hold the promise of synergistic efficacy, particularly as ferroptosis induction gains traction as a viable method to bypass drug resistance and potentiate immunotherapy. Given that silencing lncRNAs has already demonstrated feasibility in preclinical models, mapping the precise oncogenic signaling of LINC02178 could ultimately pave the way for novel, targeted interventions against refractory HPV-positive cervical cancer.

In summary, this work culminates in the development of a robust risk model anchored in FRLs—a tool that not only refines prognostic stratification for cervical cancer but also brings functionally critical molecules into sharp focus. Central to these findings is the identification of LINC02178 as a pivotal player in the HPV16-positive landscape, where it appears to fuel malignancy by actively suppressing ferroptosis and preserving the delicate redox balance tumor cells rely on for survival. Beyond individual molecular functions, we propose a novel functional link connecting HPV16 infection, LINC02178, and lipid metabolism—a regulatory network that may be contribute to in driving disease progression. In conclusion, our findings provide new insights into the mechanisms of HPV-driven carcinogenesis. These results suggest that targeting the LINC02178 axis, potentially in combination with ferroptosis inducers, could represent a viable therapeutic strategy for treating refractory, HPV-positive cervical cancer.

## 5 Conclusion

In this study, we successfully established and validated a ferroptosis-centric RSM for cervical cancer, bridging in silico transcriptomic analysis of the TCGA-CESC cohort with tangible clinical verification. Our model demonstrated a robust capacity to stratify patient prognosis, maintaining its predictive edge even when adjusted for conventional clinicopathological variables—a finding that underscores the genuine clinical utility of ferroptosis signatures in this malignancy. Central to this risk landscape is LINC02178, which emerged as a potent high-risk factor with a striking affinity for HPV16-positive environments, both in patient tissues and cell lines. Functionally, silencing this lncRNA did more than just arrest proliferation and migration; it significantly impaired the cells of their metabolic defenses, sensitizing them to ferroptosis inducers by dismantling antioxidant capacity and fueling lipid peroxidation. Collectively, these data suggest that LINC02178 plays a critical role in ferroptosis tolerance in HPV-driven cancers, likely by recalibrating lipid metabolism and redox homeostasis to favor survival. Ultimately, this work not only refines our theoretical framework for prognostic evaluation but also illuminates FRLs as promising therapeutic targets for future intervention.

## Data Availability

The datasets presented in this study can be found in online repositories. The names of the repository/repositories and accession number(s) can be found in the article/supplementary material.
